# Notes

**Published:** 1896-09

**Authors:** 


### Notes.

---

The death of Dr. Jerome Cochran, of Alabama, is announced.

---

Prof. Edw. Klebs has been elected to the Chair of Pathology in the Rush Medical College, Chicago.

---

The next meeting of the British Medical Association will be held in Montreal, Canada, in 1897.

---

The date of meeting of the Mississippi Valley Medical Association has been changed to September 15–18. Place of meeting, St. Paul, Minn.

---

The American Electro-Therapeutic Association will hold its sixth annual meeting at Boston, Mass., September 29th and 30th.

---

The twentieth annual meeting of the American Dermatological Association will be held at the Hot Sulphur Springs, Virginia, September 8th, 9th and 10th.

---

The American Association of Obstetricians and Gynecologists will be held at the Hotel Jefferson, Richmond, Va., September 22d to 24th.

---

*Moody's Medical Magazine* is the latest claimant for journalistic honors. It has no connection with the evangelist of Northport.

---

Dr. M. Ashby Purse has been elected Professor of Chemistry in the Southern Medical College, to occupy the vacancy created by the resignation of Dr. H. F. Harris. The selection is an admirable one, as Dr. Purse is especially fitted, from experience and special knowledge of this important branch, to fill the chair with great credit.

A CASE is reported of menstruation at seven days of age. The mons veneris and breasts of the infant were considerably developed.

---

DR. A. W. STIRLING has charge of the editorial department of our esteemed contemporary, THE ATLANTA MEDICAL AND SURGICAL JOURNAL, during the absence of Dr. L. B. Grandy.

---

WE call especial attention to the advertisement of Adeps Lanae, "N. W. K." This is a stable chemical compound and is unexcelled as an elegant menstruum for ointments.

---

DR. J. B. S. HOLMES has been appointed a member of the State Board of Medical Examiners in place of Dr. J. C. Olmsted, resigned.

---

CHARLES BROADWAY ROUSS, the eccentric New York millionaire, offers a contingent fee of one million dollars to any one who will restore his sight. He is suffering from atrophy of the optic nerve.

---

THE Eighth Annual Meeting of the Tri-State Medical Society of Alabama, Georgia and Tennessee, will be held in Nashville, Tuesday, Wednesday and Thursday, October 13th, 14th and 15th, 1896.

---

MESSRS. LEA BROS. & CO. announce the publication upon September 1st of a revised edition of Gray's Anatomy. The new edition is an enlargement of the old in text, and one hundred and thirty-five new engravings have been added.

---

DR. LUCIEN LOFTON has assumed control of the *Atlanta Clinic*, having purchased the interests of Drs. Champion and Childs.

